# Novel small molecules affecting cell membrane as potential therapeutics for avian pathogenic *Escherichia coli*

**DOI:** 10.1038/s41598-018-33587-5

**Published:** 2018-10-17

**Authors:** Dipak Kathayat, Yosra A. Helmy, Loic Deblais, Gireesh Rajashekara

**Affiliations:** Food Animal Health Research Program, Department of Veterinary Preventive Medicine, The Ohio State University, Wooster, OH 44691 USA

## Abstract

Avian pathogenic *Escherichia coli* (APEC), a most common bacterial pathogen of poultry, causes multiple extra-intestinal diseases in poultry which results in significant economic losses to the poultry industry worldwide. In addition, APEC are a subgroup of extra-intestinal pathogenic *E*. *coli* (ExPEC), and APEC contaminated poultry products are a potential source of foodborne ExPEC infections to humans and transfer of antimicrobial resistant genes. The emergence of multi-drug resistant APEC strains and the limited efficacy of vaccines necessitate novel APEC control approaches. Here, we screened a small molecule (SM) library and identified 11 SMs bactericidal to APEC. The identified SMs were effective against multiple APEC serotypes, biofilm embedded APEC, antimicrobials resistant APECs, and other pathogenic *E*. *coli* strains. Microscopy revealed that these SMs affect the APEC cell membrane. Exposure of SMs to APEC revealed no resistance. Most SMs showed low toxicity towards chicken and human cells and reduced the intracellular APEC load. Treatment with most SMs extended the wax moth larval survival and reduced the intra-larval APEC load. Our studies could facilitate the development of antimicrobial therapeutics for the effective management of APEC infections in poultry as well as other *E*. *coli* related foodborne zoonosis, including APEC related ExPEC infections in humans.

## Introduction

Avian pathogenic *E*. *coli* (APEC), an extra-intestinal pathogenic *E*. *coli* (ExPEC), is one of the most common bacterial pathogens affecting chickens, turkeys, and other avian species^1,2^. APEC can affect birds of all ages and in all types of production systems either as primary or secondary pathogen. Serotypes O1, O2, O8, O18, O35, O78, O109, and O115 are commonly associated with infections and among them O1, O2, and O78 constitute more than 80% of the cases^2^. APEC causes multiple extra-intestinal infections in poultry such as airsacculitis, perihepatitis, pericarditis, peritonitis, omphalitis, salphingitis, and cellulitis which subsequently leads to high morbidity and mortality (up to 20%), reduced body weight gain and egg production, and increased carcass condemnation at slaughter (up to 45%), thus resulting in severe economic losses to the poultry industry worldwide^1^.

Several studies have also reported similarities of APEC with human ExPECs such as uropathogenic *E*. *coli* (UPEC) and neonatal meningitis *E*. *coli* (NMEC) in their phylogenetic background, genome content, and virulence factors^1,3^. Thus, poultry products are considered as major reservoirs for ExPECs and the consumption of APEC contaminated poultry products is considered as potential route of foodborne ExPEC infections to humans. Further, APEC are also a source for transmission of antimicrobial resistant genes to human pathogens, including ExPECs, which makes treatment of human infections difficult^1^. Therefore, in addition to its impact on poultry health and productivity, the foodborne transmission potential of APEC to humans necessitates effective control of APEC infections in poultry.

Antimicrobial medication using tetracyclines, cephalosporins, sulfonamides, or quinolones is the major approach currently employed to reduce the incidence and mortality associated with APEC infections in poultry worldwide^4^. However, multi-drug resistant (MDR) APEC strains resistant to tetracyclines, sulfonamides, aminoglycosides, β-lactam antimicrobials, quinolones, and colistin are reported worldwide including major poultry producing countries; United States, China, Brazil, and European Union^5–8^. In addition, currently available vaccines do not provide cross protection against multiple APEC serotypes due to heterogeneity (variability in genome content) among serotypes^2,9^.

Small molecule (SM) libraries containing diverse SMs can provide the platform for novel antimicrobial discovery^10^. SMs are defined as low molecular weight (~200–500 Da), non-peptide, organic, synthetic or natural compounds with drug-like properties that can interact with biological molecules such as protein and nucleic acids and can alter their normal functions^10^. The high-throughput screening (HTS) of SM libraries can identify the SMs that can either inhibit the bacterial growth or function of key bacterial enzymes^10^. Previous studies have identified SMs having antimicrobial activity against several human and animal pathogens^11–13^.

In the current study, we screened a SM library containing 4,182 SMs to identify and characterize novel antimicrobial therapeutics against APEC. The primary screening followed by secondary assays identified seven potent SMs affecting APEC cell membrane. These SMs were effective against multiple APEC serotypes, biofilm embedded APEC, antimicrobial resistant APECs, and other pathogenic *E*. *coli* strains. These SMs showed low toxicity towards eukaryotic cells and were effective against intracellular and intra-larval APEC. Our studies could facilitate the development of novel antimicrobial therapeutics for the effective management of APEC infections in poultry and thereby also reduce human ExPEC infections and transfer of antimicrobial resistant genes.

## Methods

### Small molecule library

A pre-selected enriched SM library containing a total of 4,182 ‘yactives’ was used. This library was derived through pre-screening of 81,320 compounds^13–15^. These 81,320 compounds were initially tested for their growth inhibitory activity against *Saccharomyces cerevisiae*. Among them 7,476 compounds were found inhibiting the growth of *S*. *cerevisiae* by at least 30%, referred as ‘yactives’. From 7,476 ‘yactives’ identified, 4,182 SMs were selected using *in silico* methods (two-property filter and Naïve Bayes model) with consideration of their physicochemical properties and substructures^15^. These pre-selection procedures significantly increased the discovery of bioactive compounds in diverse model organisms (*Schizosaccharomyces pombe*, *Cryptococcus neoformans*, *E*. *coli*, *Bacillus subtilis*, *Candida albicans*, and human A549 non-small-cell lung carcinoma) in comparison to random compounds library, thus designated as pre-selected enriched library^15^. SM library was obtained from ChemBridge at 10 mM concentration dissolved in 100% dimethyl sulfoxide (DMSO) in 96 well plates and plates were stored at −80 °C until further use.

### Bacterial strains, culture conditions, and media

APEC serotypes O1, O2, and O78 were primarily used in this study and were kindly provided by Dr. Tim Johnson (University of Minnesota, Saint Paul, MN). Other APEC serotypes APEC O1-63, O2-211, O8, O15, O18, O35, O78-53, O109, and O115 were kindly provided by Drs. Lisa K. Nolan and Catherine M. Logue (University of Georgia, Athens, GA). STEC strains were kindly provided by Dr. Jeffrey T. LeJeune (The Ohio State University, Wooster, OH). Luria-Bertani (LB) broth (BD Difco) was used for routine propagation of APEC serotypes. APEC serotypes stored in 25% glycerol at −80 °C were inoculated into LB broth and grown overnight at 37 °C with shaking at 200 rpm. For screening purpose, M63 minimal media was used to grow APEC serotypes. The M63 media was prepared as described previously^16^ and the composition is listed in Table S1. The use of minimal media allows the slow APEC growth, mimics the nutrient deficient host condition, and has been shown to increase the hits rate^17^.

### Primary screening

To identify the APEC growth inhibitors, SM library was screened against APEC O78 which is one of the most frequently isolated APEC serotypes from avian colibacillosis cases^2^. One microlitre SMs (final concentration of 100 µM) were added using a slotted pin tool (V and P Scientific, San Diego, CA, USA) to the wells of the 96-well plate containing 100 µL of overnight grown 0.05 OD_600_ (7 × 10^7^ CFU/mL) adjusted APEC culture. Controls (four replicates/plate) containing 1 µL of 100% DMSO (final concentration of 1%), 1 µL chloramphenicol (CHL, #C0378 Sigma-Aldrich) (20 µg/mL), 1 µL kanamycin (KAN, #60615 Sigma-Aldrich) (50 µg/mL), and 100 µL of M63 media were included. To determine the effect of DMSO on APEC growth, antimicrobial activity of DMSO was determined at different concentrations (1% to 32%) in a separate experiment. Plate was then incubated at 37 °C for 12 h in Sunrise - Absorbance microplate reader (Tecan Group Ltd. San Jose, CA) with kinetic OD_600_ measurement every 30 mins. The quality of screening was assessed by calculating the Z′-score as described previously^18^. The growth inhibition of APEC was calculated by using the formula as previously described^13,14^. The SMs inhibiting at least 80% of the APEC growth were selected as primary hits. Culture from wells considered as hits were subsequently subcultured on LB agar plate to determine the bactericidal effect (no APEC recovered on plating following exposure to SM); these cidal SMs were selected for further studies.

### MIC and MBC determination

SMs were two-fold serially diluted from 200 µM to 6.25 µM to determine their MIC and MBC as described previously^19^. One microlitre SM of each concentration was transferred to each well of a 96-well plate containing 100 µL of the 0.05 OD_600_ adjusted APEC O78 culture in M63 media. Growth was monitored in Sunrise - Absorbance microplate reader as described above. MIC was indicated by lowest concentration of SM with non-elevated OD_600_ measurement. MBC was determined by absence of APEC growth on LB agar plate following subculture. In addition, MIC and MBC of cidal SMs were also determined as described above against multiple APEC serotypes (O1, O2, O8, O15, O18, O35, O109, and O115) that are commonly associated with colibacillosis cases^2^ to determine their spectrum of activity. Two independent experiments were conducted. The activity of cidal SMs were also tested at 100 µM in M63 media against Shiga toxin-producing (STEC) O157 and O26 strains (Table S2).

### Effect against antimicrobial resistant APECs

Initially, antimicrobial susceptibility profile was established for all the tested APEC serotypes using cation-adjusted Muller-Hinton broth (CAMHB) micro-dilution method according to clinical and laboratory standards institute (CLSI) guidelines^20^. *E*. *coli* ATCC 25922 was used as quality control strain. Four antimicrobials; ampicillin (AMP, #A9518 Sigma-Aldrich), ciprofloxacin (CIP, #17850 Sigma-Aldrich), colistin (CST, C4461 Sigma-Aldrich), and tetracycline (TET, T7660 Sigma-Aldrich) that are currently used in poultry industry and belonging to different classes of antimicrobials; penicillins, quinolones, polymixins, and tetracyclines, respectively were evaluated for susceptibility according to their MIC breakpoints for resistance (AMP ≥ 16 µg/mL, CIP ≥ 4 µg/mL, CST ≥ 4 µg/mL, and TET ≥ 16 µg/mL)^21^. To determine the effect against antimicrobial resistant APECs, the MIC and MBC of cidal SMs were compared between the antimicrobial susceptible and resistant APEC serotypes.

### Effect against beneficial microbes

SMs were screened against different beneficial microbes to determine their specificity as described previously^13^. The beneficial microbes used in this study along with their culture requirements are listed in Table S2. SMs were added at 100 µM to 100 µL of 0.05 OD_600_ adjusted bacterial cultures in specific growth media in 96-well plate, and plate was incubated under indicated conditions. The specific growth media and conditions required for beneficial microbes limited the use of minimal media. Following incubation, endpoint OD_600_ was measured and cultures from the wells with non-elevated OD_600_ were plated on selective agar plates to determine the bactericidal effect.

### Effect against biofilm embedded APEC

The effect of cidal SMs against biofilm embedded APEC was determined using MBEC High-throughput (HTP) assay (Innovotech Inc., AB, Canada)^22^. Briefly, 150 µL of 0.05 OD_600_ adjusted APEC O78 culture was aliquoted into each well of the MBEC device containing polystyrene pegs and incubated at 37 °C for 36 h in LB media under stationary condition. After biofilm formation, the pegs were washed to remove loosely adherent planktonic bacteria, transferred to new 96-well plate, and challenged with different concentrations of SMs (0.5X, 1X, 2X, 4X, and 8X MIC) in 200 µL M63 media. The plate was incubated in the dark for 18 h at 37 °C with rotation at 110 rpm. The DMSO (1%) and M63 media were used as positive and negative controls, respectively. Following incubation, MIC of SMs in challenged plate was recorded. The SMs exposed pegs were then transferred to a new 96-well plate containing PBS and sonicated for 30 mins (Aquasonic ultrasonic cleaner, VWR) to disrupt the biofilm. The sonicated suspensions were ten-fold serially diluted and plated on LB agar plate. Biofilm embedded APEC bacteria were enumerated and minimum biofilm eradication concentration (MBEC) of SMs were determined as described previously^22^. Two independent experiments were conducted.

### Antimicrobial resistance studies

To evaluate APEC O78 potential to acquire resistance against cidal SMs, single step (lethal dose) and sequential passage (sub-lethal dose) resistance assays were performed in M63 media as described previously^13,14,19^. Briefly, for single step resistance assay, SMs were mixed with 1.5 mL of molten M63 agar at a final concentration of 2X MBC and transferred to wells of a sterile 24-well plate. Concentration of 2X MBC was used since it has been previously reported that the MIC/MBC of antimicrobials are higher in solid media compared to liquid media^23,24^. Fifty microlitres of overnight grown APEC O78 (~10^9^ CFU) culture was plated over the solidified SM amended M63 agar. The plate was incubated for 15 days in the dark at 37 °C. After 15 days, any colonies that grew on the agar were assessed for resistance by determining the MIC and MBC as described above.

For sequential passage resistance assay, SMs were added at a final concentration of 0.75X MIC (concentration that allows at least 70% growth inhibition) to the 100 µL of the 0.05 OD_600_ adjusted APEC O78 culture in M63 media in a 96-well plate. The plate was then incubated in the dark at 37 °C with shaking at 150 rpm for 18 h. After the first incubation, bacterial pellet was resuspended in a fresh M63 media amended with 0.75X MIC of each SM and grown as above. This procedure was repeated 14 times. Following 15 passages, susceptibility (MIC and MBC) of APEC to SMs was determined as described above. DMSO (1%), 20 µg/mL CHL, 50 µg/mL KAN, and M63 media were included as controls in both the assays. Experiments were conducted in duplicate wells.

### Confocal and scanning electron microscopy

Confocal microscopy was used for bacterial cytological profiling (BCP) to identify the cellular pathways targeted by SMs as described previously^25^. Briefly, 100 µL of logarithmic-phase APEC O78 culture grown in M63 media was treated with 2X MBC of SMs and incubated at 37 °C for 2 h with shaking at 200 rpm. After incubation, treated cultures were centrifuged, washed, and resuspended in 100 µL PBS. FM4-64 (#T13320 Molecular Probes/Invitrogen) (1 µg/mL) and SYTO-9 (#S34854 Molecular Probes/Invitrogen) (5 µM) were added to the bacterial cultures and incubated for 45 mins at room temperature with shaking at 150 rpm. Cultures were then centrifuged, washed, and resuspended in PBS to 1/10^th^ volume of the original cultures. Three microlitres of concentrated bacterial cultures were transferred onto an agarose pad containing 1.2% agarose and 20% LB medium. Microscopy was performed using Leica TCS SP6 confocal scanning microscope (Excitation/emission (nm); FM4-64 (515/640), SYTO-9 (485/498) and images were analyzed using ImageJV1.50.

The SMs treated APEC O78 cultures prepared above were also processed for scanning electron microscopy (SEM) as described previously^26^. SEM was performed for representative SMs (possessing similar structure and BCP). Briefly, one volume of bacterial culture was mixed with one volume of fixative (3% glutaraldehyde, 1% paraformaldehyde in 0.1 M potassium phosphate buffer, pH 7.2), and incubated at 4 °C overnight. The fixed bacterial cells were then centrifuged for 5 mins at 1,200 × g, washed twice with PBS, and resuspended in 1% osmium tetroxide for 1 h at room temperature in the dark, followed by serial dehydration of the sample in ethanol and platinum splatter-coating. Visualization and imaging of the sample was performed using a Hitachi S-4700 scanning electron microscope.

### Membrane permeability assays

Membrane permeability assays (crystal violet (CV) uptake and loss of 260/280 nm absorbing materials) were conducted as described previously^27^. For CV uptake assay, APEC O78 culture grown in M63 media was adjusted to 0.2 OD_600_ (~10^8^ CFU/mL) and treated with 2X MBC of SMs for 30 mins followed by incubation for 10 mins with 10 µg/mL CV. For the loss of 260/280 nm absorbing materials assay, APEC O78 cultures adjusted to 1.0 OD_600_ (~10^9^ CFU/mL) in M63 media were treated with 2X MBC of SMs for 1 h. DMSO (1%) and 0.25 M ethylenediaminetetraacteic acid (EDTA, #8991-01 JT Baker) were used as negative and positive controls, respectively in both the assays. CV uptake was measured using the formula: (OD DMSO − OD SM/OD DMSO × 100). Two independent experiments in duplicates were conducted.

### Cytotoxicity of SMs to chicken and human cells

The cytotoxicity of cidal SMs to human Caco-2 and chicken HD11 cells were evaluated using Pierce Lactate Dehydrogenase (LDH) Cytotoxicity Assay Kit (Pierce, Thermo Scientific, Rockford, IL, USA) as previously described^13,14^. Cytotoxicity was measured at OD 680 nm and 490 nm after exposing cultured epithelial and macrophage cells to 200 µM of SMs for 24 h. 10X LDH provided in the kit was used as positive control. Two independent experiments with triplicate wells in each experiment were conducted.

### Hemolytic activity of SMs to chicken RBCs

The hemolytic activity of cidal SMs to chicken RBCs was evaluated as previously described^13^. Hemolysis was determined at OD 540 nm after exposing 10% RBCs suspension to 200 µM of SMs for 1 h. 0.1% Triton X-100 (#BP151-100, Fisher Scientific) was used as positive control. Two independent experiments with triplicate wells in each experiment were performed.

### Effect of the SMs on intracellular survival of APEC in phagocytic and non-phagocytic cells

Intracellular survival assay was conducted as described previously^28,29^ to determine the effect of cidal SMs on APEC survival in phagocytic (HD11, THP-1) and non-phagocytic (Caco-2) cells. Briefly, mid-logarithmic phase grown APEC O78, O1, and O2 were washed and adjusted to 1 × 10^7^ CFU/mL in cell culture incomplete media (no FBS and antibiotics). One-hundred microlitres adjusted APEC suspension was added at multiplicity of infection (MOI) 10 to wells of 96-well cell culture plate containing cultured macrophage (HD11, THP-1) and epithelial cells (Caco-2) and incubated for 1 h and 3 h, respectively. For APEC O1, invasion time was reduced by 3 times in all cell types as APEC O1 was found with significantly *(P* < *0*.*01)* higher invasiveness compared to O78 and O2 (Fig. S1). After incubation, cells were washed and treated with 150 µg/mL gentamicin (#157100164, Fisher Scientific) for 1 h to kill extracellular APEC. The cells were then washed, replenished with incomplete media containing different concentrations (0.5X, 1X, 2X, and 4X MIC) of SMs, and incubated for 6 h. The cells were then lysed with 100 µL of 0.1% Triton X-100 for 5 mins, serially diluted, and plated on LB agar plate to enumerate viable bacteria. The intracellular bacteria in SMs treated wells were compared with DMSO (1%) treated wells. Two independent experiments in duplicate wells for each concentration of SMs were conducted.

### Toxicity and efficacy of SMs in wax moth (*Galleria mellonella*) larvae

For toxicity evaluation, *G*. *mellonella* larvae (fifth instar) were inoculated with 12.5 µg of SMs (50 mg/kg body wt.) through last pro-leg using PB600-1 repeating dispenser (Hamilton, Reno, NV) attached to insulin syringe (31 gauge, 8 mm needle length) (ReliOn^®^, Bentonville, AR). For the inoculation, SMs were diluted in buffer mix containing 30% DMSO and 10 mM MgSO_4_ (#r-375-25, CQ Concepts) as described previously^30^. Post-inoculation, larvae were placed inside sterile petri dishes and incubated up to 72 h in the dark at 37 °C and larval survival was monitored every 12 h. Non-treated larvae, larvae treated with the buffer mix, and larvae treated with CHL (75 mg/kg body wt.; dose sufficient to clear APEC infection in larvae) were used as controls.

For SMs efficacy testing, larvae were first injected with SMs mixed in buffer through the left hind pro-leg at dose rate as described above and incubated for 2 h at 37 °C. Then, larvae were infected with 6 × 10^4^ CFU of Rif^r^ APEC O78 in 10 mM MgSO_4_ on the right hind pro-leg. Rif^r^ APEC O78 was generated by plating APEC on LB agar plate containing 50 µg/mL rifampicin for specific monitoring of APEC population inside the larvae. SMs displayed identical MIC and MBC to Rif^r^ APEC O78 as the wild-type (Fig. S2). Infection dose of Rif^r^ APEC O78 to larvae was identified based on preliminary study (Table S3). Infected larvae inoculated with buffer mix were used as positive control whereas larvae inoculated with CHL were used as negative control. Post-inoculation, larval survival was monitored as above. For the quantification of APEC load inside the dead and live larvae, larvae from SMs treated and control groups were surface sterilized with 70% ethanol and homogenized in PBS. The suspension was ten-fold serially diluted and plated on MacConkey agar (#R453802, Fisher Scientific) plates supplemented with 50 µg/mL of rifampicin. The plates were then incubated overnight at 37 °C and APEC load was enumerated. Each experiment was repeated twice using larvae (n = 20) obtained in different batches.

### Statistical analysis

The statistical significance of the effect of SMs in reducing biofilm embedded and intracellular APEC was determined by one-tailed Student’s t-test *(P* < *0*.*01)*. The significance of CV uptake and increase of OD 260 and 280 nm absorbing bacterial supernatants in SMs treated samples was statistically analyzed by one-tailed Student’s t-test *(P* < *0*.*05)*. Kaplan-Meir survival curves were generated using GraphPad Prism V.5 and were statistically analyzed by log-rank test *(P* < *0*.*05)*. APEC load inside the SMs treated and control larvae were analyzed by one-way ANOVA tukey’s test using GraphPad Prism V.5 *(P* < *0*.*05)*. APEC load inside the live and dead larvae were statistically compared using one-tailed Student’s t-test *(P* < *0*.*05)*. Correlation (r) between the larval survivability and APEC load was calculated using Microsoft Excel 2010.

## Results

### Primary screening identified 40 SMs inhibitory to APEC O78 growth

**Figure 1:**
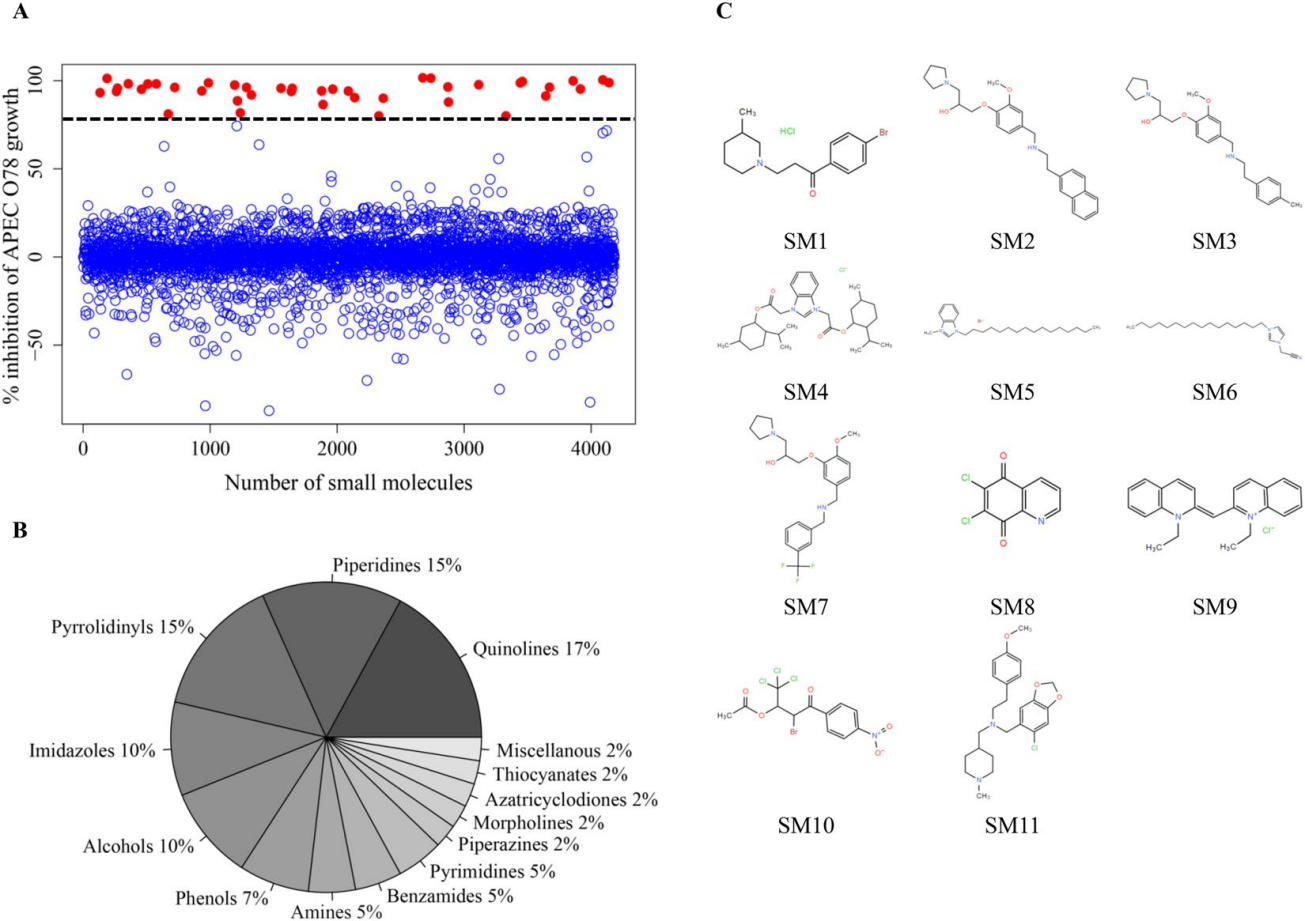
(**A**) HTS of 4,182 SMs to identify novel anti-APEC SMs. The growth inhibitory activity was assessed by incubating APEC O78 in the presence of SMs (100 µM) followed by kinetic OD_600_ measurement for 12 h using Sunrise - Absorbance microplate reader. A total of 40 SMs (~1%) inhibited more than 80% growth of APEC O78 (indicated by dashed black line) and were considered as primary hits (red filled circles). Only 11 SMs (0.25% of total SMs) displayed bactericidal activity and were selected for further studies. (**B**) Chemical groups of 40 primary hits. Chemical groups are shown as percentage of total hits. (**C**) Chemical structures of 11 selected anti-APEC bactericidal SMs identified in this study; Piperidines (SM1, SM11), Pyrrolidinyls (SM2, SM3, SM7), Imidazoles (SM4, SM5, SM6), Quinolines (SM8, SM9), Miscellaneous (SM10).

In the primary screening, 4,182 SMs were assessed for the growth inhibition of APEC O78 using 100 µM of SMs. A total of 40 SMs (hits) inhibited the APEC growth more than 80% (Fig. 1A). One percent DMSO was used as control, which did not affect the growth of APEC, only DMSO concentration of 8% and above was inhibitory to APEC (Fig. S3). The average Z′- score for the HTS assay was 0.85 which is more than the Z′- score (>0.5) for a successful HTS assay^18^. The majority of hits belonged to quinolines (~17%) followed by piperidines (~15%), pyrrolidinyls (~15%), and imidazoles (~10%) (Fig. 1B). Among these hits, 11 SMs (SM1-SM11) (Fig. 1C) exhibited bactericidal effect. These 11 SMs belonged to pyrrolidinyls (SM2, SM3, and SM7), imidazoles (SM4–SM6), quinolines (SM8 and SM9), piperidines (SM1 and SM11), and miscellaneous (SM10) group of chemical compounds (Fig. 1C).

### Seven SMs possessed MIC as low as 25 µM

**Figure 2:**
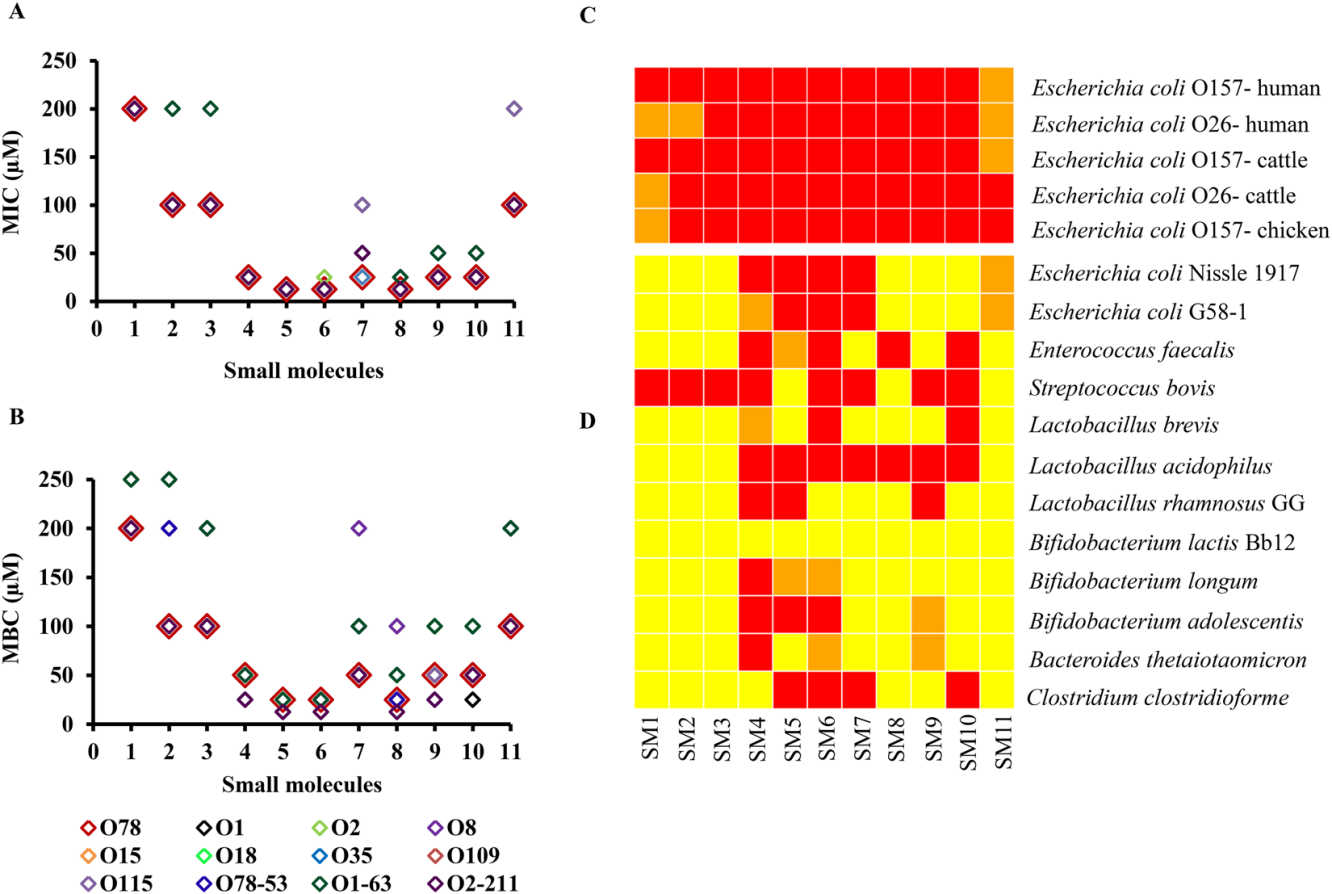
MIC (**A**) and MBC (**B**) of 11 cidal SMs against different APEC serotypes. The MIC and MBC of SMs against APEC O78 are shown with large red open quadrangles to compare MIC and MBC of SMs against other APEC serotypes. (**C**,**D**) Heat map displaying the effect of 11 cidal SMs to STEC strains (**C**) and commensal/beneficial microbes (**D**). SMs were tested at 100 µM. Red box indicates the cidal activity; orange box indicates the static activity, and yellow box indicates no effect of SMs against tested bacteria.

Of the 11 SMs, seven SMs (SM4–SM10) possessed MIC ranging from 12.5 to 25 µM (Fig. 2A, Table S4). The other three SMs (SM2, SM3, and SM11) possessed MIC of 100 µM. SM1 though was bactericidal to APEC at 100 µM in the primary screening, upon re-synthesis and subsequent testing it showed cidal activity only at 200 µM. Piperidines (SM1 and SM11) and pyrrolidinyls (SM2, SM3, and SM7) group of SMs were effective only at high MIC (25 µM–200 µM), whereas quinolines (SM8 and SM9) and imidazoles (SM4-SM6) groups of SMs were effective at low concentration (12.5–25 µM). Most of the SMs had MBC twice the MIC except, SM1, SM2, SM3 and SM11; these SMs had MBC identical to MIC (Fig. 2B, Table S4).

### SMs are effective against multiple APEC serotypes, antimicrobial resistant APECs, and STEC strains

Anti-APEC therapeutics with broad APEC activity is needed due to multiple and genetically heterogeneous APEC serotypes implicated in field infections^2^. All 11 SMs inhibited the growth of all tested APEC serotypes, with MIC & MBC mostly equivalent to those of APEC O78 (Fig. 2A,B, Table S4). The MIC’s of antibiotics against *E*. *coli* ATCC 25922 were; AMP: 8 µg/mL, CIP: < 0.03125 µg/mL, CST: 1 µg/mL, TET: 0.5 µg/mL which were within the CLSI suggested range. The APEC serotypes tested were resistant to TET (O1, O1-63, O2, O2-211, O8, O15, O78, O78-53), AMP (O78, O1, O2, O109, O115), CST (O1-63, O2-211), and CIP (O18) (Table S5). Antimicrobial therapeutics with efficacy against multi-antibiotic resistant pathogens is crucial to combat antimicrobial resistance^31,32^. The antimicrobial efficacies of 11 SMs are equivalent between the susceptible and resistant APEC serotypes (Fig. 2A,B, Table S4). This indicated that these SMs could be applicable to control APEC serotypes that are resistant to antimicrobials that are currently used to treat APEC infections in poultry. All 11 SMs were inhibitory at 100 µM against STEC O157 and O26 strains isolated from different sources (Fig. 2C). Most of the SMs (SM3–SM10) were bactericidal at 100 µM for these STEC strains; however, SM1, SM2, and SM11 were not cidal at 100 µM (Fig. 2C). STEC strains are associated with human illnesses and are a public health concern^33^. These results suggest potential applicability of these SMs to manage the *E*. *coli* related foodborne zoonosis.

### Six SMs affected limited number of commensal/probiotic bacteria

The use of non-specific and broad spectrum antimicrobials have effect on beneficial microbes leading to the disturbance of microbiota which renders host susceptible to infections by pathogens^34^. In addition, use of broad spectrum antimicrobials also enriches the abundance of resistant microorganisms and resistant genes in the microbiota which in turn could foster the antimicrobial resistance problem^35^. Six of these SMs (SM1-SM3, SM8, SM9, and SM11) exerted least effect on beneficial microbes; having cidal activity against one to three of the 12 commensal/probiotic bacteria tested at 100 µM (Fig. 2D). Whereas, three SMs (SM4-SM6) belonging to imidazoles group were bactericidal to most of the tested probiotics/commensals. Even though piperidines (SM1, SM11) and pyrrolidinyls (SM2, SM3) group of SMs possessed higher MICs than other SMs, they displayed more specific activity against APEC. Interestingly, most of the SMs (SM1–SM3, SM8, SM9, and SM10) did not have effect on *E*. *coli* Nissle 1917 and *E*. *coli* G58-1 and none of the SMs exerted effect on *Bifidobacterium lactis* Bb12 (Fig. 2D). Overall, *Lactobacillus brevis*, *Lactobacillus rhamnosus* GG, *Bifidobacterium lactis* Bb12, and *Bacteroides thetaiotaomicron* are the microbes least affected by these SMs.

### Nine SMs eradicated biofilm embedded APEC

Bacterial biofilms confers increased resistance to antimicrobials thus it is difficult to treat biofilms protected bacteria^36^. Of the 11 selected SMs, nine (SM1-SM7, SM9, and SM11) SMs possessed MBEC ranging 0.5X to 4X MIC in MBEC HTP assay (Table 1). Imidazoles (SM4-SM6) and pyrrolidinyls (SM2, SM3, and SM7) SMs were effective in eradicating biofilm embedded APEC bacteria at 0.5X MIC to 2X MIC. SM1 and SM11 possessed MBEC of 4X MIC. SM8 (t = −12.56) and SM10 (t = −22.08) significantly (*P* < *0*.*01*, Student’s t-test) reduced the biofilms embedded APEC at 1X MIC; however, they were not able to eradicate biofilm embedded APEC even at 8X MIC.

**Table 1:** Effect of 11 cidal SMs against biofilm embedded and intracellular APEC bacteria.

	MBEC^†^ (µM)	SMs intracellular APEC clearance concentration^††^ (µM)								
		O78			O2			O1		
		Caco-2	HD11	THP-1	Caco-2	HD11	THP-1	Caco-2	HD11	THP-1
SM1	400^d^	200^a^	200^a^	200^a^	>400	200^a^	200^a^	>400	400^b^	400^b^
SM2	50^b^	100^a^	100^a^	100^a^	>200	200^b^	100^a^	>200	200b	200^b^
SM3	50^b^	100^a^	100^a^	100^a^	>200	200^b^	100^a^	>200	200^b^	200^b^
SM4	25^a^	50^b^	50^b^	50^b^	100^c^	50^b^	100^c^	100^c^	50^b^	>100
SM5	25^c^	50^c^	50^c^	50^c^	100^d^	50^c^	50^c^	100^d^	100^d^	100^d^
SM6	12.5^a^	50^c^	50^c^	50^c^	100^d^	50^c^	50^c^	100^d^	100^d^	100^d^
SM7	50^b^	100^c^	50^b^	50^b^	100^c^	100^c^	25^a^	100^c^	100^c^	>100
SM8	>100	50^c^	50^c^	50^c^	50^c^	50^c^	50^c^	50^c^	50^c^	50^c^
SM9	50^b^	50^b^	100^c^	50^b^	100^c^	100^c^	50^b^	100^c^	100^c^	50^b^
SM10	>200	100^c^	50^b^	100^c^	100^c^	100^c^	50^b^	100^c^	>100	>100
SM11	400^d^	100^a^	100^a^	100^a^	200^b^	200^b^	100^a^	>200	200^b^	>200

^†^SMs MBEC; ^a^0.5X MIC, ^b^1X MIC, ^c^2X MIC, ^d^4X MIC. ^**††**^SMs intracellular APEC clearance concentration; ^a^1X MIC, ^b^2X MIC, ^c^4X MIC, ^d^>4XMIC. SMs MBEC and intracellular APEC clearance concentration with “>” arrow indicates SMs not able to eradicate completely the biofilm embedded APEC bacteria or SMs not able to completely clear intracellular APEC up to the concentrations tested.

### No resistance was detected in APEC O78 to SMs

Identical MBCs were observed when APEC O78 was grown in sub-lethal (0.75X MIC) doses of SMs in liquid media for 15 overnight passages (90 generations) (Fig. S4). After 15 days of incubation of APEC O78 on solid media amended with a 2X MBC of SMs, no resistant colonies were observed. These results suggest that the 11 SMs were less likely to induce resistance in APEC O78; however, more in-depth characterization of resistance is needed for future development and application of these SMs in the field.

### SMs exhibited antimicrobial activity by affecting APEC cell membrane

**Figure 3:**
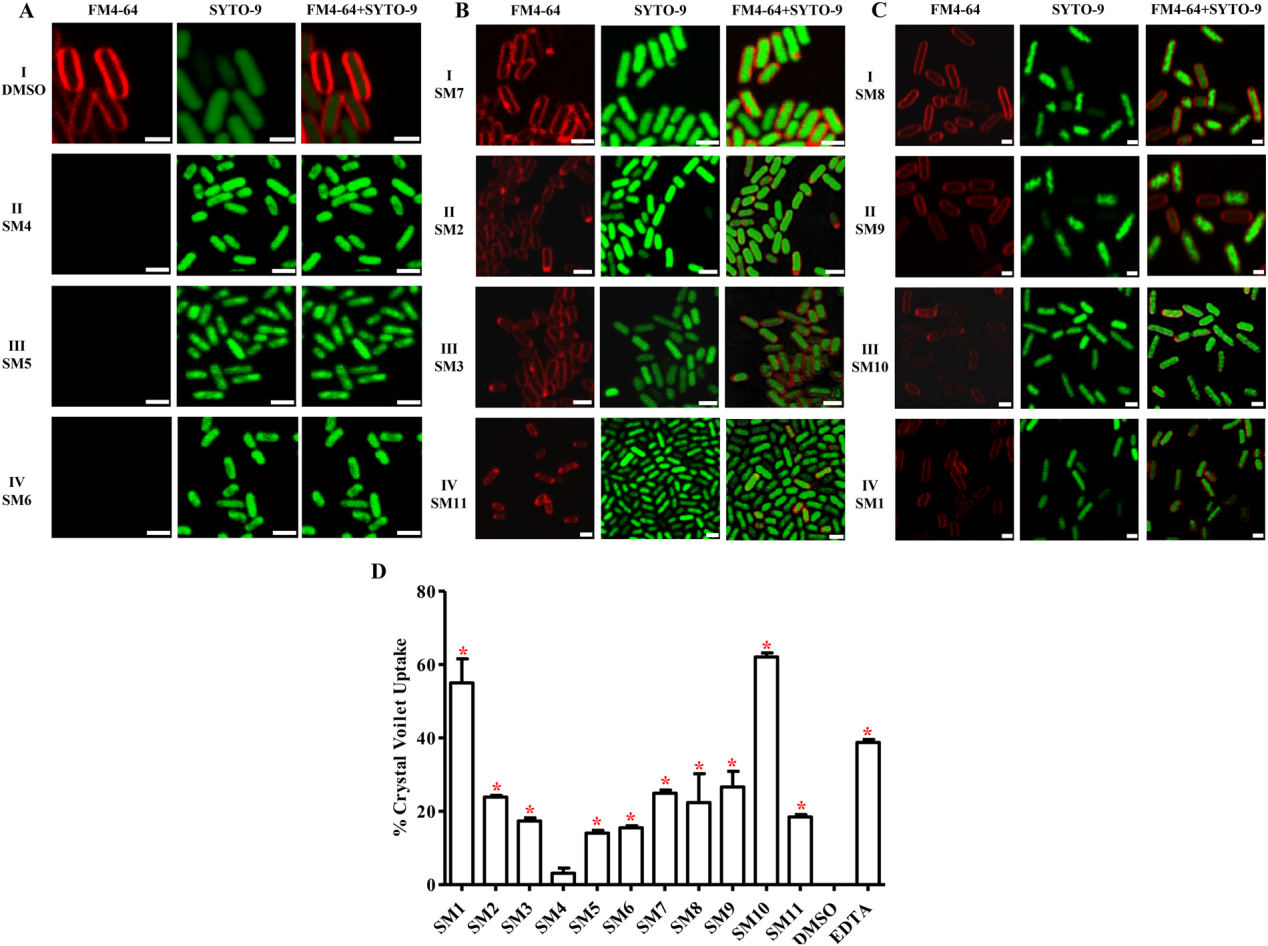
Confocal microscopy images of SMs treated APEC bacteria. Logarithmic phase grown APEC O78 cultures were treated with 2X MBC of SMs followed by the staining with FM4-64 (membrane stain; red colored) and SYTO-9 (nucleic acid stain; green colored). (**A**;I) DMSO treated APEC bacteria showing stained membrane and nucleic acid, (**A**;II–IV**)** SM4, SM5, and SM6 showing absence of FM4-64 stained membrane (membrane disruptors), (**B**;I–IV) SM7, SM2, SM3, and SM11 showing bright red foci at random positions throughout the cell indicating membrane defects (membrane pore formers), (**C**;I–IV) SM8, SM9, SM10, and SM1 showing filamentous or short rods (spheroplasts) morphology (PG synthesis inhibitors). Bars: 1 µM. (**D**) CV uptake assay. APEC O78 was treated with 2X MBC of SMs, incubated with crystal violet, and absorbance of the supernatants was measured at OD 590 nm. Uptake of CV was significantly higher in SMs treated samples compared with DMSO treated sample and was similar to EDTA treated sample. *P < *0*.*05*.

BCP is regarded as a rapid and powerful approach to identify the cellular pathways affected by different antibacterials based on the cytological changes induced by SMs^25^. Our study revealed that the 11 cidal SMs are likely to functions by either disrupting cell membranes or producing membrane defects or inhibiting cell wall peptidoglycan (PG) synthesis (Fig. 3). DMSO treated APEC bacteria showed stained membrane and nucleic acid (Fig. 3A;I). Imidazoles SMs (SM4–SM6) are likely to disrupt the cell membrane which is similar to polymixins mechanism of action (MOA)^37^ and is evident by the absence of FM4-64 stained bacterial cell membrane (Fig. 3A;II–IV). Pyrrolidinyls SMs (SM2, SM3, and SM7) and SM11 are likely to produce membrane defects by forming pores as similar to those induced by daptomycin^38^ and macrocyclic peptide JB-95^39^ MOA which is evident by the presence of FM4-64 stained bright foci or protrusions at random positions on the cell (Fig. 3B;I–IV). Quinolines SMs (SM8 and SM9) along with SM1 and SM10 produced either filamentous or short rods (spheroplasts) morphology (Fig. 3C;I–IV) of APEC which is similar to ampicillin and cephalexin antibiotics^25,40^, these antibiotics inhibit the synthesis of cell wall PG.

Membrane permeability assays revealed SMs affecting the cell membrane integrity. SMs as well as EDTA treatment significantly (*P* < *0*.*05*, Student’s t-test, SM1, t = 8.36; SM2, t = 52.15; SM3, t = 20.24; SM5, t = 19.04; SM6, t = 28.10; SM7, t = 29.15; SM8, t = 2.84; SM9, t = 6.26; SM10, t = 58.15; SM11, t = 27.13; EDTA, t = 47.38) increased the uptake of crystal violet (CV) ranging 3–62% (Fig. 3D) except, SM4 (*P* = *0*.*07*, t = 2.22). CV can penetrate the cells with altered membrane permeability^41^. SMs and EDTA treatment also significantly (*P* < *0*.*05*, Student’s t-test) increased the 260 (SM1, t = 31.23; SM4, t = 9.32; SM5, t = 31.23; SM6, t = 18.95; SM7, t = 19.07; SM8, t = 31.23; SM9, t = 19.07; SM10, t = 11.77; SM11, t = 31.23; EDTA, t = 31.23) or 280 nm (SM1, t = 21.88; SM2, t = 25.99; SM3, t = 13.87; SM4, t = 3.62; SM5, t = 3.63; SM6, t = 3.86; SM7, t = 9.97; SM8, t = 18.59; SM9, t = 11.03; SM10, t = 8.34; SM11, t = 11.93) absorbing materials in the treated supernatants in comparison to DMSO treatment (Table 2). Intracellular constituents such as DNA, RNA, proteins can be leaked through permeable membrane^41^.

**Table 2:** SMs treatment induced leakage of 260 and 280 nm absorbing material.

	Absorbance (nm)	
	260	280
SM1	4.00^a^	3.08^a^
SM2	3.71	2.86^a^
SM3	3.77	2.84^a^
SM4	3.91^a^	2.69^a^
SM5	4.00^a^	2.67^a^
SM6	3.99^a^	2.69^a^
SM7	3.91^a^	2.70^a^
SM8	4.00^a^	2.82^a^
SM9	3.99^a^	2.78^a^
SM10	3.98^a^	2.73^a^
SM11	4.00^a^	2.89^a^
EDTA	4.00^a^	2.60
DMSO	3.77	2.58

SMs treatment induced OD increment was compared with DMSO treatment. ^a^*P* < *0*.*05*.

**Figure 4:**
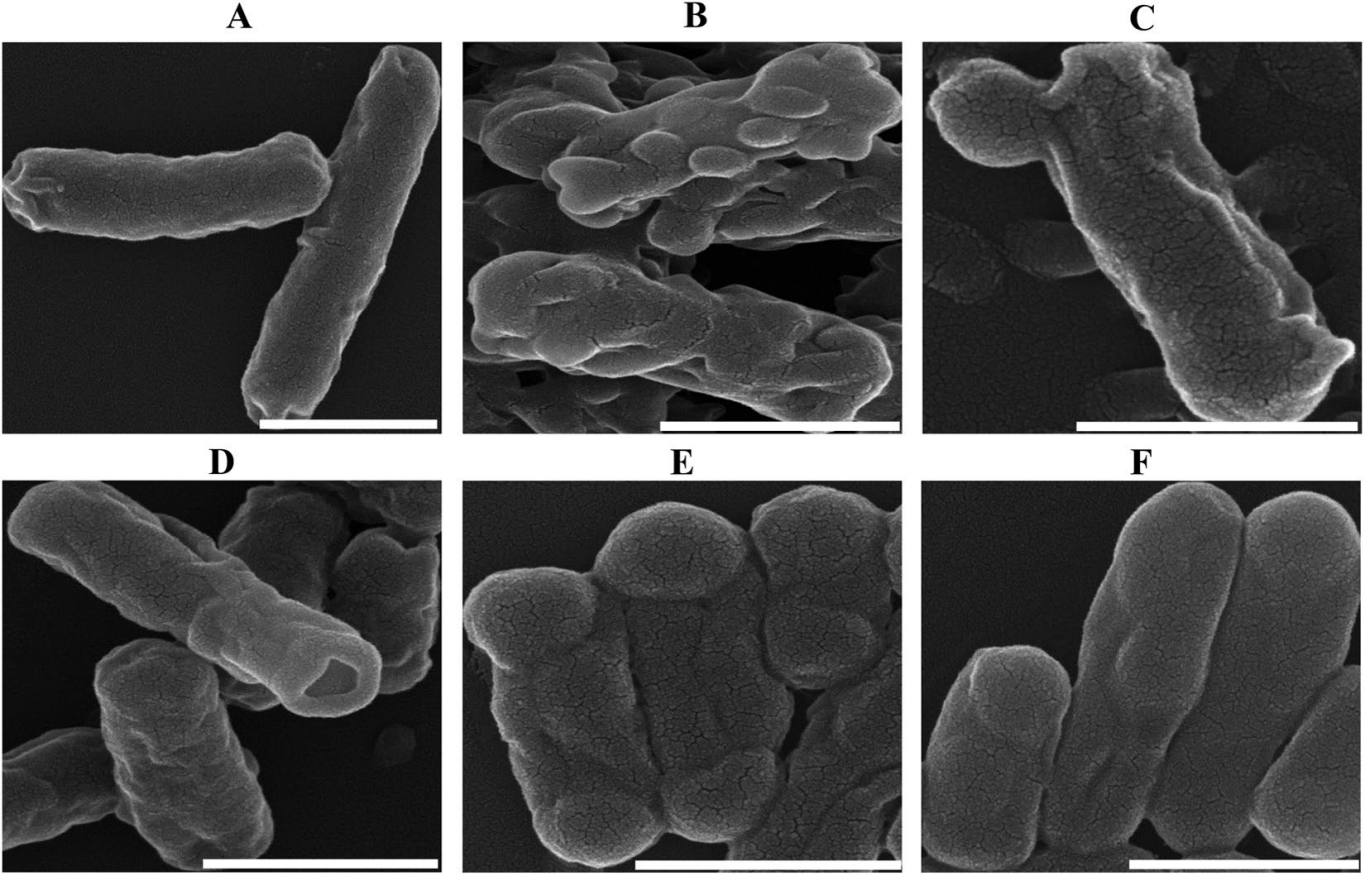
Representative SEM images of SMs treated APEC bacteria. (**A**) DMSO treated bacteria showing less wrinkled smooth surface cells measuring 1–2 µM, (**B**) SM4 - showing severe wrinkling and multiple blebbing throughout the cells (membrane disruptors), (**C**,**D**) SM3 and SM7 - showing blebbing or pore at single cell pole, respectively (membrane pore formers), (**E**,**F**) SM8 and SM10 - showing shortened cells (~0.5 µM) and blebbing at cell poles (PG synthesis inhibitors). Bars: 1 µM.

SEM results further supported the cell membrane affecting mode of action of SMs. SEM images suggest that SMs treatment produced membrane wrinkling, blebbing/vesicle-like structures, and pores (Fig. 4) which are the characteristics morphology induced by membranes acting antibiotics and several other antimicrobial agents^42–47^. DMSO treated APEC bacteria showed very few wrinkled smooth surfaced cells measuring 1–2 µM (Fig. 4A). The frequency and sites of blebbing and severity of wrinkling differed between the SM treatments. SM6 (Fig. 4B) produced more severe wrinkling and multiple blebbing throughout the cells. SM3 and SM7 formed blebbing and pore at single cell pole, respectively (Fig. 4C,D). SM8 and SM10 produced distinct morphology than other SMs with shortened cells (~0.5 µM) and blebbing at both cell poles (Fig. 4E,F) which is similar to ampicillin induced cells morphology^40^. Even though, these studies showed SMs inducing APEC membrane alterations; however, the observed changes in membrane morphology could be either direct or indirect effects of the SMs.

### SMs showed minimal toxicity toward chicken and human cells

**Figure 5:**
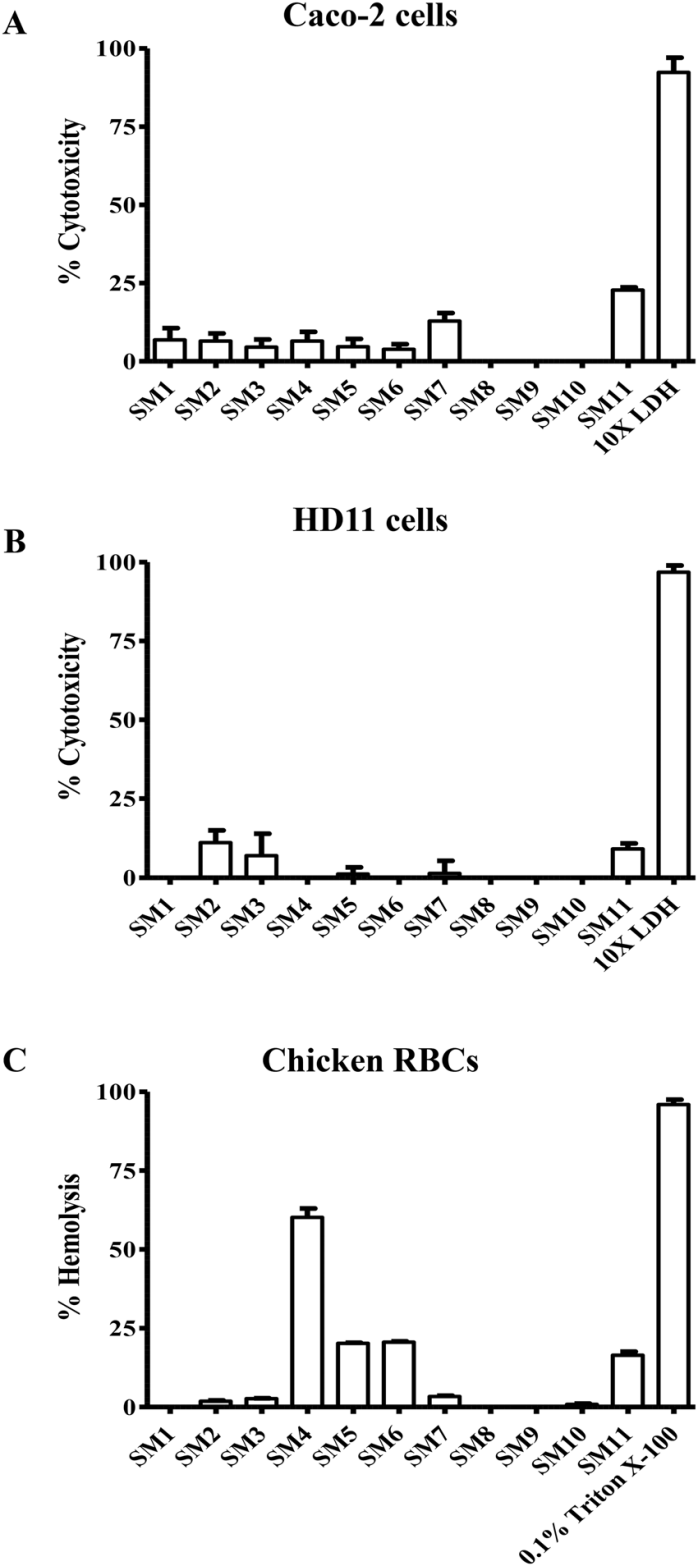
Cytotoxicity of 11 cidal SMs to (**A**) Caco-2 and (**B**) HD11 cells. Toxicity was assessed by measuring the LDH released from lysed cells after incubation with 200 µM concentration of SMs for 24 h. 10X LDH was used as positive control. (**C**) Hemolytic activity of SMs to chicken RBCs. 10% washed RBCs were incubated with 200 µM of SMs for 1 h and the hemoglobin released from lysed RBCs was measured. 0.1% Triton X-100 was used as positive control.

Based on LDH assay, most of the SMs possessed least cytotoxicity (<10%) on Caco-2 (Fig. 5A) and HD11 cells as compared to 10X LDH (Fig. 5B), except SM11. Among 11 cidal SMs, four SMs (SM4-SM6, and SM11) caused hemolysis (20–60%) to RBCs (Fig. 5C) while the rest of the SMs displayed <10% of hemolysis as compared to 0.1% Triton X-100.

### SMs reduced intracellular APEC in phagocytic and non-phagocytic cells

The fimbria mediated initial APEC adhesion and *OmpA*/*IbeA* mediated invasion into the cells facilitate APEC to survive intracellularly in phagocytic and non-phagocytic cells of the host and is an important aspect of APEC pathogenesis^48^. Therefore, the administered antimicrobial therapeutics must be able to permeate and act inside the APEC infected cells. After 6 h of treatment, SMs significantly (*P* < *0*.*01*, Student’s t-test, see Table S6 for t values) reduced intracellular APEC O78, O2, and O1 in infected Caco-2, HD11, and THP1 cells at varying concentrations (0.5 × –2X MIC) with maximal reduction (3–5 log; 100% clearance) of intracellular APEC O78, O2 and O1 at concentration less than or equal to 4X MIC (Table 1), except SM5 and SM6. SM5 and SM6 possess very high LogP (SM5: 8.75, SM6: 10.19) compared to other SMs; high LogP values cause poor permeation and absorption of drugs through the membranes^49^. Among 11 SMs, SM4–SM10 were effective in clearing intracellular APEC O78, O2, and O1 at concentration less than or equal to 100 µM for most of the cases; whereas, SM1–SM3 and SM11 were effective only at concentration equal or above 100 µM (Table 1). Interestingly, higher concentrations of SMs were needed to clear intracellular APEC O1 followed by O2 and O78 (Table 1) which may be due to greater invasion and survival of O1 serotype inside the cells (Fig. S1). The serotype O1 is reported to carry *IbeA (*invasin) and *Iss* (increased serum survival) genes more frequently compared to O78 and O2^50,51^ which might contribute for better invasion and survival. SM8 was the most effective SM in clearing intracellular APEC with complete clearance at concentration less than or equal to 50 µM (Table 1). Overall, SM4, SM7, SM8, SM9, and SM10 were the most effective SMs in clearing intracellular APEC serotypes in all tested cells.

### SMs showed low toxicity toward wax moth larvae, extended the larval survival, and reduced the APEC load inside the larvae

**Figure 6:**
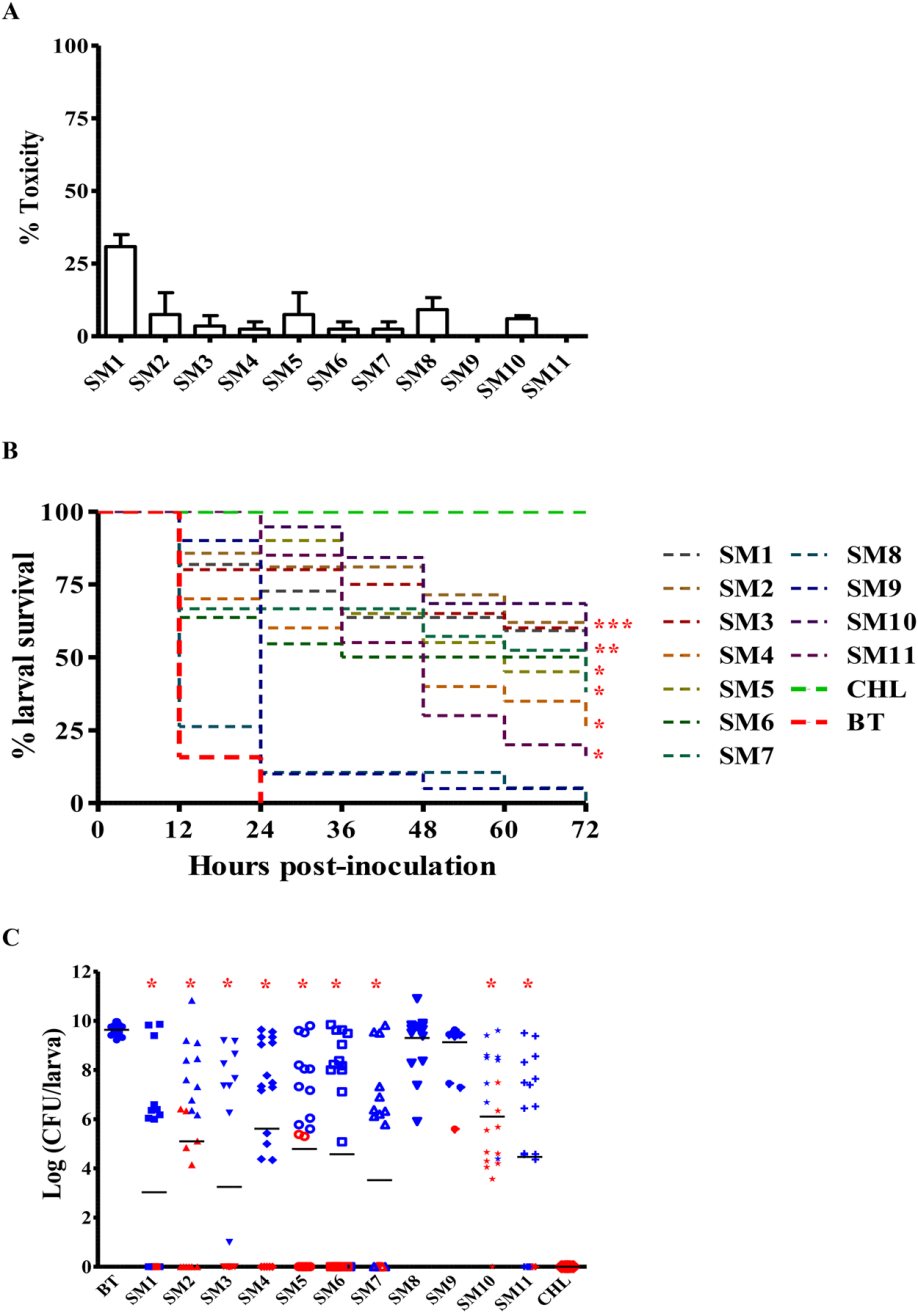
(**A**) Toxicity of SMs to wax moth (*G*. *mellonella*) larvae (n = 20). SMs were injected to larvae (12.5 µg/larva) and larval survival was monitored for 72 h. (**B**) Kaplan-Meir survival curves of APEC infected larvae treated with SMs (12.5 µg/larva). Larvae (n = 20) were injected with SMs 2 h before infection with the Rif^r^ APEC O78 and larval survival was monitored for 72 h. Survival curves of SMs treated larvae were compared with buffer mix treated larvae. ^*^P < *0*.*05*. (**C**) Scatter plot displaying APEC load in SMs treated larvae. APEC load was quantified from dead larvae (blue symbols) collected every 12 h and live larvae (red symbols) collected at 72 h post-infection. APEC load of SMs treated larvae were compared with buffer mix treated larvae. ^*^P < *0*.*05*. BT- buffer mix treated; CHL- chloramphenicol treated.

The wax moth larval model is increasingly used in the recent years as an alternative to mammalian model to study bacterial pathogenesis and antimicrobial drug testing^52^. Except SM1, rest of the SMs showed less toxicity (<10%) to larvae (Fig. 6A).

Most of the SMs (SM1- SM7, and SM10 - SM11) significantly (*P* < *0*.*05*, log-rank test, SM1, χ^2^ = 22.80; SM2, χ^2^ = 22.53; SM3, χ^2^ = 21.71; SM4, χ^2^ = 15.03; SM5, χ^2^ = 28.76; SM6, χ^2^ = 12.60; SM7, χ^2^ = 14.60; SM10, χ^2^ = 39.29; SM11, χ^2^ = 36.88) extended the survival of infected larvae (Fig. 6B). The larva survival rate was higher in the SM treated groups as compared to the buffer mix treated group. In the treated groups, 15–55% of infected larvae survived even at 72 h post-infection, while all larvae injected with buffer mix died by 24 h. All of the SMs which extend the survival of infected larvae also significantly (*P* < *0*.*05*, tukey’s test, SM1, q = 9.28; SM2, q = 6.30; SM3, q = 8.77; SM4, q = 5.52; SM5, q = 6.65; SM6, q = 7.11; SM7, q = 8.49; SM10, q = 4.84; SM11, q = 7.09) reduced the APEC load (3–6 log) inside the larvae (Fig. 6C). Live larvae had significantly low (*P* < *0*.*01*, Student’s t-test, t = −18.04) APEC load (1 log on an average) in comparison to dead larvae (7 log on an average) which also correlated with the survival of the larvae (r = 0.7).

### Structure-activity relationship analysis

**Figure 7:**
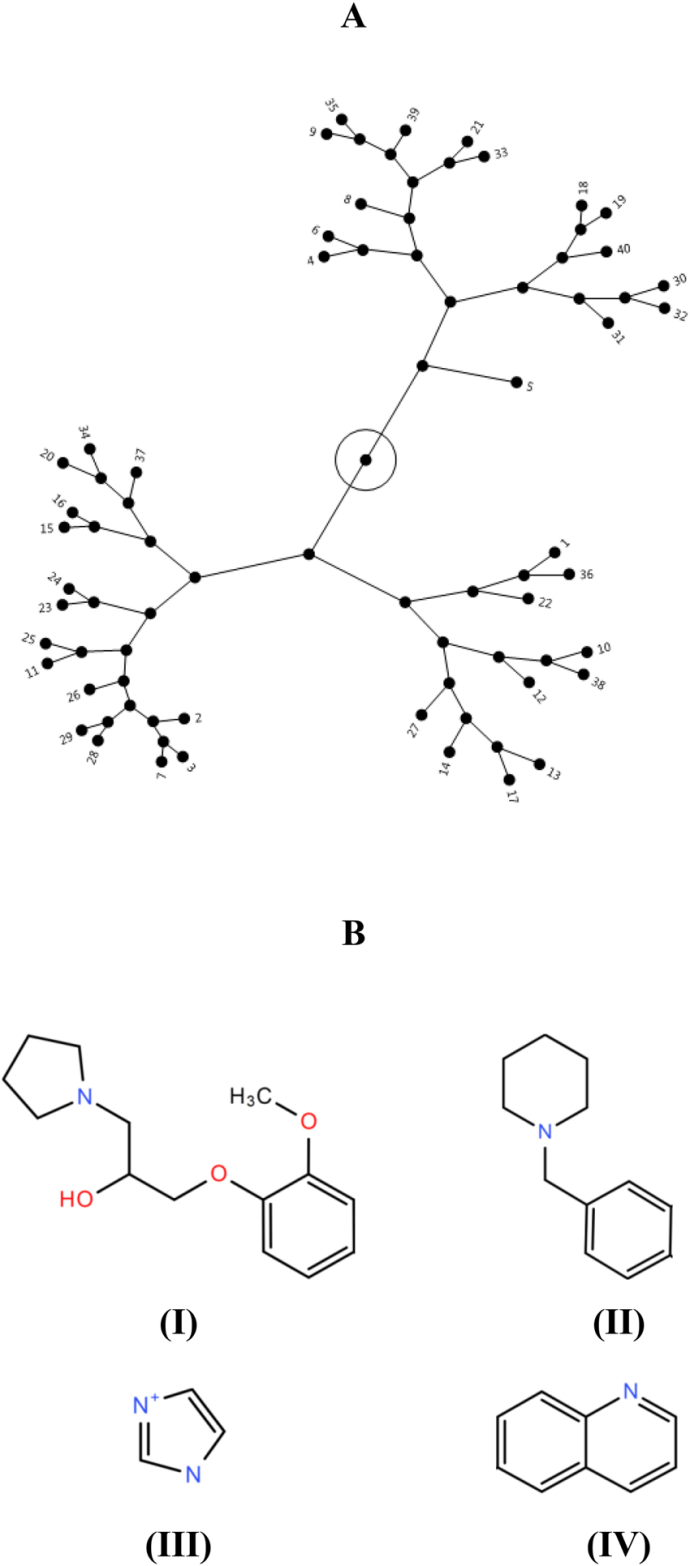
(**A**) Constellation plot depicting structural clustering (2D-Tanimoto) of 40 primary hits generated based on structural similarity between hits. Structural similarity scores were retrieved from PubChem database and plot was generated using JMP software. Hits were clustered into three major clusters. The 11 selected bactericidal SMs are labelled 1–11. SMs belonging to same chemical groups were mostly clustered together, (**B**) Common scaffolds identified in anti-APEC SMs in this study. (I) pyrrolidinyl, (II) piperidine, (III) imidazole, and (IV) quinoline.

Structural clustering of identified hits based on their 2D-Tanimoto similarity showed imidazole (SM4-SM6) and quinoline (SM8, SM9) SMs structurally more close with nitrogen-containing aromatic ring in common which could contribute for their lower MIC and MBC in comparison to pyrrolidinyl (SM2, SM3, SM7) and piperidine (SM1, SM11) SMs (Figs 1C, 2A,B and 7A). Among the pyrrolidinyl hits, hits with additional benzene ring in the pyrrolidine scaffold showed bactericidal activity (Fig. 1C) whereas hits without benzene ring showed only bacteriostatic activity (not shown). In addition, among the bactericidal pyrrolidinyl SMs (SM2, SM3, SM7), SM7 possesses trifluoro group which could contribute to its bactericidal activity at lower concentration in comparison to SM2 and SM3 (Fig. 1C). The hydroxy-methoxybenzyl group is absent in bactericidal piperidine SMs (SM1, SM11) (Fig. 1C) in comparison to bacteriostatic piperidine hits (not shown). SM10, which belongs to miscellaneous group contains trihalogen (trichloro) group (Fig. 1C) as similar to SM7 which could contribute to its bactericidal activity at lower concentration (Fig. 2B).

## Discussion

APEC is responsible for severe economic losses to the poultry industry worldwide^1,2^ and is also regarded as a potential source of human ExPECs^1^. Effective novel control methods are needed because of the limitations associated with current control methods^5,9^. Anti-APEC SMs identified in our study are diverse in their structures with three major clusters based on structural similarity (Fig. 7A) and contained pyrrolidinyl, piperidine, imidazole, and quinoline scaffolds (Fig. 7B). In previous studies, chemical compounds having the piperidine and pyrrolidine rings were reported with antimicrobial activity against several infectious pathogens^53,54^. Likewise, imidazoles and the 2,4,5 –trisubstituted imidazole derivatives were regarded as versatile and promising antimicrobials as well as sensitizers of MDR pathogens^55–57^. Quinolinedione derivatives were also reported as potent antimicrobial agents against Gram negative and positive bacteria^58^. Though compounds belonging to same chemical groups were reported with antimicrobial activity against several pathogenic bacteria including *E*. *coli* in earlier studies, there is no previous report of the antimicrobial activity of the compounds identified in our study except, SM9. SM9 was reported to inhibit *E*. *coli* BW25113 growth (57%) at 100 µM (https://pubchem.ncbi.nlm.nih.gov/bioassay/710). These identified anti-APEC scaffolds could facilitate the development of antimicrobial therapeutics to control APEC infections in poultry.

Anti-APEC SMs identified in our study affect the APEC cell membrane. Bacterial cell membranes are regarded as promising targets for discovery of new antimicrobial therapeutics and to combat antimicrobial resistance^59^. Membrane affecting antimicrobials are most likely to act by disrupting membrane architecture and functional integrity^59^ which is supported by our confocal and SEM images and membrane permeability assays (Figs 3 and 4). Under confocal microscopy, SMs treated APEC bacteria showed membrane disrupted morphology along with formation of membrane defects throughout the cell (Fig. 3A,B,C). The disruption of the cell membrane and formation of membrane defects could subsequently leads to leakage of cell contents, loss of membrane potential, and eventual cell death^59^. Further, SEM analysis revealed that SMs treatment induced membrane wrinkling, blebbing/vesicle-like structures, and pores (Fig. 4) which consequently could impair the cell membrane integrity leading to cell death^38^. The membrane defects caused by SMs resembles to those caused by already known membrane acting antibiotics such as polymixins (SM4-SM6)^37^, daptomycin (SM2, SM3, SM7, and SM11)^38^, ampicillin/cephalexin (SM1, SM8-SM10)^25,40^, and several other antimicrobial peptides^32,42–47^. Polymixins disrupt the outer membrane integrity of Gram negative bacteria by forming the blebs on the surface of the bacterium^31^. Daptomycin induces holes in the membrane leading to a breach in the cell membrane and subsequent cell death by forming membrane blebs^38^. Multiple antimicrobial peptides such as Human α-defensin 5 (HD5), gramicidin S, peptidyl-glycylleucine-carboxyamide (PGLa), cathelicidins, lactoferricin, and human epididymis 2 (HE2) protein isoforms damage the bacterial cell membrane by forming the blebs^32,43–46^. Peptoids, an alternative to antimicrobial peptides, damage the membrane of *E*. *coli* by forming membrane blebs^47^. Sericin, a soluble silk glue protein exhibits antibacterial activity against *E*. *coli* by inducing blebbing of the membrane^42^. Furthermore, previous studies have also shown that quinoline, imidazole, piperidine, and pyrrolidine compounds possessing antibacterial activity by affecting the bacterial cell membranes^60–63^ which is consistent with our findings.

The SMs identified in our study are effective against multiple APEC strains, STEC strains as well as antimicrobials resistant strains (Fig. 2) which might be explained by their membrane affecting mode of action. Antimicrobials that target the cell membranes exhibit broad spectrum of activity and are being used to control MDR bacteria such as ESKAPE pathogens^31,32^, methicillin resistant *Staphylococcus aureus* (MRSA)^60^; therefore, SMs identified in our study could be used to treat APEC infections caused by antimicrobial resistant strains. Membrane affecting antimicrobials also have a low potential for development of resistance mostly due to their effect on multiple targets^59^. Consistent with low resistance acquisition of membrane affecting antimicrobials, no resistant APECs were isolated *in vitro* in our study which could makes these SMs as emergency antimicrobials in APEC outbreaks situation. Permeability of APEC cell membrane is also impaired following SMs treatment (Fig. 3D). Thus, the incorporation of these SMs in therapy could enhance the uptake or penetration of antibiotics that have intracellular targets^59^ or could interact synergistically with other membrane affecting antibiotics^64^. In fact, several SMs significantly decreased the MBC of TET, CST, and CIP that are commonly used to treated APEC infection in poultry (unpublished data). As a result, combining these SMs could increase the activity of antibiotics or reduce the amount of antibiotics needed, and by consequence, could attenuate the development of antimicrobial resistance associated with APEC in poultry.

Most of the identified SMs, especially imidazoles (SM4-SM6) and pyrrolidinyls (SM2, SM3, SM7), eradicated biofilm embedded APEC even at 0.5X to 2X MIC (Table 1) which could be due to low molecular wt. of SMs allowing better penetration inside the biofilms^36^ or could be due to inherent biofilm dispersal/disruption activity of imidazoles^65^ or anti-biofilm activity of pyrrolidinyls^66^. Membrane affecting antimicrobials have capacity to act against slow-growing or dormant bacteria as well as on biofilms^59^. APEC can form biofilms in poultry facilities such as in water lines and drinker systems^67^ and are difficult to eradicate by common disinfectants and antimicrobials. Therefore, the SMs identified in our study could be used to eradicate biofilm embedded APEC in poultry facilities; thereby reducing the incidence and occurrence of APEC infections in poultry farms. SM8 and SM10, which are effective against planktonic and intracellular bacteria even at low concentration (Fig. 2, Table 1) showed decreased effectivity towards biofilm embedded APEC which could be due to restricted penetration of SMs inside the biofilm or could be due to binding with biofilm matrix^68^. Additionally, these SMs contain chlorine atoms in common; bacterial biofilms are increasingly resistant to chlorine treatment^69^.

We used the “yactives” library under the premise that compounds with this property may be enriched for bioactivity against non-yeast-based chemical screens as was originally shown by Wallace *et al*.^15^. Previous screen in yeast was conducted using higher concentration (200 µM) of SMs as compared to our screening (100 µM) (Fig. 1). At 200 µM, the SMs were considered “yactives” if they inhibited the yeast growth at least by 30%; however, the most potent SMs identified in our study showed almost 100% inhibition of APEC at lower concentration (8-16x less; 12.5–25 µM) (Fig. 2); therefore, we anticipated least effect of these SMs on eukaryotic cells. Indeed, most of the identified SMs (SM1-SM3, SM7-SM10) showed less toxicity towards chicken and human cells (Fig. 5). The toxicity of the membrane affecting antimicrobials depends upon the membrane organization and its lipids composition and proportion^59^. Both epithelial and macrophage cells membrane contain phosphatidylcholine (PC) as major phospholipid^59^; however, bacterial cell membrane is rich in phospholipids such as phosphatidylglycerol (PG), phosphatidylethanolamine (PE) and cardiolipin (CL) which makes membrane affecting antimicrobials selectively toxic to bacterial cells^70^. RBCs also contain phosphatidylethanolamine (PE) phospholipid in their membrane similar to bacterial cell membrane^71^; this similarity could attribute toxicity of some of the SMs (SM4-SM6, and SM11) to RBCs. The presence of cyclohexyl and/or benzodioxol groups in SM4 and SM11 could contribute for their relatively high toxicity (Fig. 1C)^72,73^. These SMs were however not toxic to wax moth larvae which could be due to cellular analogy of wax moth larva to mammals (epithelial cells of larva gut similar to intestinal cells of mammals)^74^. Consistent with wax moth studies, no negative impact of SM5 and SM6 on chicken health and performance was observed in our pilot experiment (data not shown). Further, most of the SMs identified exerted no effect on tested Gram positive bacteria such as *Lactobacillus* and *Bifidobacterium* (Fig. 2D). The use of rich media however could attribute to lesser effect of SMs to beneficial microbes. The Gram positive and Gram negative bacteria also have different composition and relative amounts of lipids in their membranes^75^. Gram positive bacterial genera such as *Clostridium*, *Lactobacillus*, and *Bifidobacterium* are the predominant commensals of the poultry gut microbiota^76^. Thus, we expect lesser impact on the microbiota of the chickens treated with these SMs^34^. Further, LGG and Bb12, widely used probiotics^77^ are unaffected by these SMs, they could be combined with these SMs to enhance the probiotics control of APEC infections in poultry.

The treatment with most of the identified SMs cleared the intracellular APEC in the infected phagocytic and non-phagocytic cells (Table 1); similar effect within the host cells could help to ameliorate APEC pathogenicity^28,48^. Consistent with the SMs intracellular clearance of APEC, SM1–SM3, SM4–SM6, SM7, SM10, and SM11 treatment significantly reduced the APEC load inside the wax moth larvae. The lesser efficacies of SM8 and SM9 in wax moth larvae in comparison to cultured epithelial and macrophage cells could be due to interaction with host immune components of wax moth larvae such as antimicrobial peptides or due to production of drug degradative enzymes^78^. Wax moth larvae possess complex innate immune system similar to mammals and several studies including studies in ExPEC have reported the similar results between wax moth and mammalian models^30,79^. Besides, wax moth larval model has been frequently used to evaluate the efficacy and toxicity of antimicrobial agents^52^. Therefore, the efficacy of these SMs in cultured infected cells and wax moth larvae may suggest their therapeutic efficacy in chickens.

In conclusion, this study had identified seven novel SMs (SM3, SM5-SM10) (Fig. S5) as potentially effective and safe (two foremost parameters of any therapeutic drug) anti-APEC therapeutics for poultry. These SMs function through affecting APEC cell membrane and can also be combined with other anti-APEC strategies such as antibiotics and probiotics. Our future studies will focus on testing SMs efficacy in chickens, identifying SMs molecular targets to define their modes of action, and also to develop these SMs to control *E*. *coli* related foodborne zoonosis including APEC related ExPEC infections in humans.

## Electronic supplementary material

Supplemental Tables and Figures

## Data Availability

All data generated or analysed during this study are included in this published article (and its Supplementary Information files).
