# Supplementary material for: Novel small molecules affecting cell membrane as potential therapeutics for avian pathogenic Escherichia coli
Source: Sci Rep. 2018 Oct 17;8:15329. doi: 10.1038/s41598-018-33587-5 (PMC6193035; doi:10.1038/s41598-018-33587-5)
Supplement: Supplementary file 1 — Supplemental Tables and Figures [file 41598_2018_33587_MOESM1_ESM.pdf]

Novel small molecules affecting cell membrane as potential therapeutics for avian pathogenic *Escherichia coli*

Dipak Kathayat, Yosra A. Helmy, Loic Deblais, and Gireesh Rajashekara*.

Table S1. Composition of M63 minimal media.

5X M63 stock (per litre)	
$(NH_4)_2SO_4$	10 g
KH_2PO_4	68 g
$FeSO_4.7H_2O$	2.5 g
Adjust to pH 7 with NaOH	
Autoclave (121^0C, 15 mins)	
**1X M63 working solution (per litre)**	
5X M63	200 mL
1M $MgSO_4.7H_2O$	1 mL
20% Glucose	10 mL
0.5% Thiamine	0.1 mL
20% Casamino Acids	5 mL
Sterilized Milipore Water	783.9 mL

Table S2. List of beneficial microbes and pathogenic STEC strains used in this study along with their growth media and conditions.

Microbes	Bacterial spp.	Media	Culture conditions	Reference/Source
Beneficial	*Enterococcus faecalis*	MRS broth	37^0C, anaerobic, 16-18 h	David Francis, SDSU
	Streptococcus bovis	MRS broth	37^0C, anaerobic, 16-18 h	David Francis, SDSU
	Lactobacillus brevis	MRS broth	37^0C, anaerobic, 1-2 days	David Francis, SDSU
	Lactobacillus acidophilus	MRS broth	37^0C, anaerobic, 1-2 days	David Francis, SDSU
	Lactobacillus rhamnosus GG	MRS broth	37^0C, anaerobic, 1-2 days	ATCC, Manassas, VA, USA
	Bifidobacterium longum	MRS broth + 0.05% Cysteine	37^0C, anaerobic, 24 h	David Francis, SDSU
	Bifidobacterium adolescentis	MRS broth + 0.05% Cysteine	37^0C, anaerobic, 24 h	David Francis, SDSU
	Bifidobacterium lactis Bb12	MRS broth + 0.05% Cysteine	37^0C, anaerobic, 24 h	Christian Hansen Ltd., Hørsholm, Denmark
	Clostridium clostridioforme	BHI broth + 5% Horse blood	37^0C, anaerobic, 6-7 days	David Francis, SDSU
	Escherichia coli Nissle 1917	LB broth	37^0C, aerobic, 10-12 h, 200 rpm	Dr. Ulrich Sonnenborn, Ardeypharm GmbH, Herdecke, Germany
	Escherichia coli G58-1	LB broth	37^0C, aerobic, 10-12 h, 200 rpm	David Francis, SDSU
	Bacteroides thetaiotaomicron	MRS broth	37^0C, anaerobic, 4-5 days	David Francis, SDSU
Pathogenic	*Escherichia coli* O157- human	LB broth	37^0C, aerobic, 10-12 h, 200 rpm	Jeffrey T. LeJeune, FAHRP,OSU
	Escherichia coli O26- human	LB broth	37^0C, aerobic, 10-12 h, 200 rpm	Jeffrey T. LeJeune, FAHRP,OSU
	Escherichia coli O157 – cattle	LB broth	37^0C, aerobic, 10-12 h, 200 rpm	Jeffrey T. LeJeune, FAHRP,OSU
	Escherichia coli O157 –chicken	LB broth	37^0C, aerobic, 10-12 h, 200 rpm	Jeffrey T. LeJeune, FAHRP,OSU
	Escherichia coli O26 -chicken	LB broth	37^0C, aerobic, 10-12 h, 200 rpm	Jeffrey T. LeJeune, FAHRP,OSU

Table S3. Standardization of Rif^r APEC O78 infection dose for wax moth larvae.

	Larva mortality (%)					
Rif^r O78 inoculum (CFU/larva)	12 h	24 h	36 h	48 h	60 h	72 h
4.25×10^3	40	70	80	80	90	90
4.25×10^4	70	90	100	-	-	-
$8.50 \times 10^{4\dagger}$	90	100	-	-	-	-
4.25×10^5	100	-	-	-	-	-

†inoculum caused 100% mortality within 24 h and provided the basis for selection of infection dose to wax moth larvae.

Table S4. Minimum inhibitory concentration (MIC) and minimum bactericidal concentration (MBC) of 11 selected SMs against different APEC serotypes.

	**Small molecules (MIC/MBC) (µM)**										
	**1**	**2**	**3**	**4**	**5**	**6**	**7**	**8**	**9**	**10**	**11**
**O78**	200/200	100/100	100/100	25/50	12.5/25	12.5/25	25/50	12.5/25	25/50	25/50	100/100
**O1**	200/250	200/250	100/200	25/50	12.5/12.5	12.5/25	50/100	12.5/25	25/50	25/25	100/100
**O2**	200/200	200/250	100/200	25/50	12.5/25	25/25	25/50	25/50	25/50	25/50	100/200
**O8**	200/200	100/200	100/100	25/25	12.5/12.5	12.5/12.5	100/200	25/100	25/50	50/50	200/200
**O15**	200/250	100/100	100/100	25/25	12.5/12.5	12.5/25	50/50	12.5/50	25/100	50/100	100/100
**O18**	200/200	100/200	100/100	25/50	12.5/12.5	12.5/25	50/100	12.5/50	25/100	25/100	200/200
**O35**	200/200	100/200	100/200	25/25	12.5/25	12.5/25	25/50	12.5/25	25/50	25/50	100/200
**O115**	200/200	200/200	100/100	25/25	12.5/12.5	12.5/25	100/100	12.5/50	25/50	25/50	200/200
**O109**	200/200	100/200	100/200	25/25	12.5/12.5	12.5/12.5	100/100	12.5/25	25/50	25/50	200/200
**O78-53**	200/200	100/200	100/200	25/50	12.5/12.5	12.5/25	50/50	12.5/25	25/25	25/50	100/200
**O1-63**	200/250	200/250	200/200	25/50	12.5/25	12.5/25	50/100	25/50	50/100	50/100	100/200
**O2-211**	200/200	100/100	100/100	25/25	12.5/12.5	12.5/12.5	50/50	12.5/12.5	25/25	25/50	100/100

Table S5. Antimicrobial susceptibility profiles of APEC serotypes.

	MIC (µg/mL)			
	**Ampicillin**	**Ciprofloxacin**	**Colistin**	**Tetracycline**
**O78**	>64 (R)	0.125	0.25	32 (R)
**O1**	>64 (R)	<0.03125	0.5	>64 (R)
**O2**	>64 (R)	<0.03125	0.5	>64 (R)
**O8**	4	<0.03125	1	>64 (R)
**O15**	2	<0.03125	1	>64 (R)
**O18**	4	4 (R)	1	2
**O35**	2	<0.03125	1	1
**O109**	>64 (R)	<0.03125	0.5	1
**O115**	>64 (R)	<0.03125	1	>64 (R)
**O78-53**	4	<0.03125	0.5	>64 (R)
**O1-63**	4	<0.03125	4 (R)	16 (R)
**O2-211**	4	<0.03125	4 (R)	>64 (R)

Resistant APEC serotypes are depicted as (R).

Table S6. t-statistic values measuring the effect of SMs to clear intracellular APEC.

	Caco-2			HD11			THP-1		
	**O78**	**O2**	**O1**	**O78**	**O2**	**O1**	**O78**	**O2**	**O1**
**SM1**	-8.08[a]	-15.12[b]	-11.33[b]	-10.48[a]	-6.98[a]	-9.11[a]	-15.39[a]	-139.34[b]	-18.03[a]
**SM2**	-8.20[a]	-10.13[b]	-6.68[a]	-8.47[a]	-5.28[a]	-9.76[a]	-7.96[a]	-138.34[b]	-20.52[a]
**SM3**	-14.33[a]	-6.97[b]	-24.49[c]	-11.43[a]	-7.34[a]	-8.79[a]	-11.55[a]	-3.22[a]	-10.20[a]
**SM4**	-16.22[a]	-14.45[c]	-49.89[c]	-8.44[a]	-7.64[a]	-5.77[b]	-4.50[a]	-4.33[b]	-18.46[b]
**SM5**	-5.38[b]	-14.75[c]	-4.13[c]	-7.75[b]	-10.60[b]	-9.59[b]	-10.56[b]	-11.12[c]	-24.69[b]
**SM6**	-14.23[c]	-17.80[c]	-12.51[c]	-9.17[b]	-11.64[b]	-10.08[b]	-7.94[b]	-10.12[c]	-18.77[b]
**SM7**	-8.96[b]	-21.23[c]	-3.50[b]	-7.87[b]	-4.22[b]	-9.48[b]	-6.63[b]	-5.22[b]	-41.02[b]
**SM8**	-3.46[b]	-6.65[b]	-43.60[b]	-7.76[b]	-7.24[b]	-8.50[b]	-10.56[b]	-4.78[b]	-30.91[b]
**SM9**	-8.98[b]	-29.80[c]	-9.42[c]	-9.76[c]	-12.72[c]	-5.00[b]	-11.55[b]	-3.89[b]	-84.43[b]
**SM10**	-10.16[b]	-9.39[b]	-3.41[b]	-116.35[c]	-7.41[b]	-7.77[b]	-12.76[b]	-7.11[b]	-16.69[b]
**SM11**	-8.99[b]	-12.16[b]	-14.32[b]	-5.48[a]	-6.47[a]	-16.30[b]	-16.87[a]	-9.22[a]	-19.74[b]

[a]0.5X MIC, [b]1X MIC, [c]2XMIC.

Fig. S1

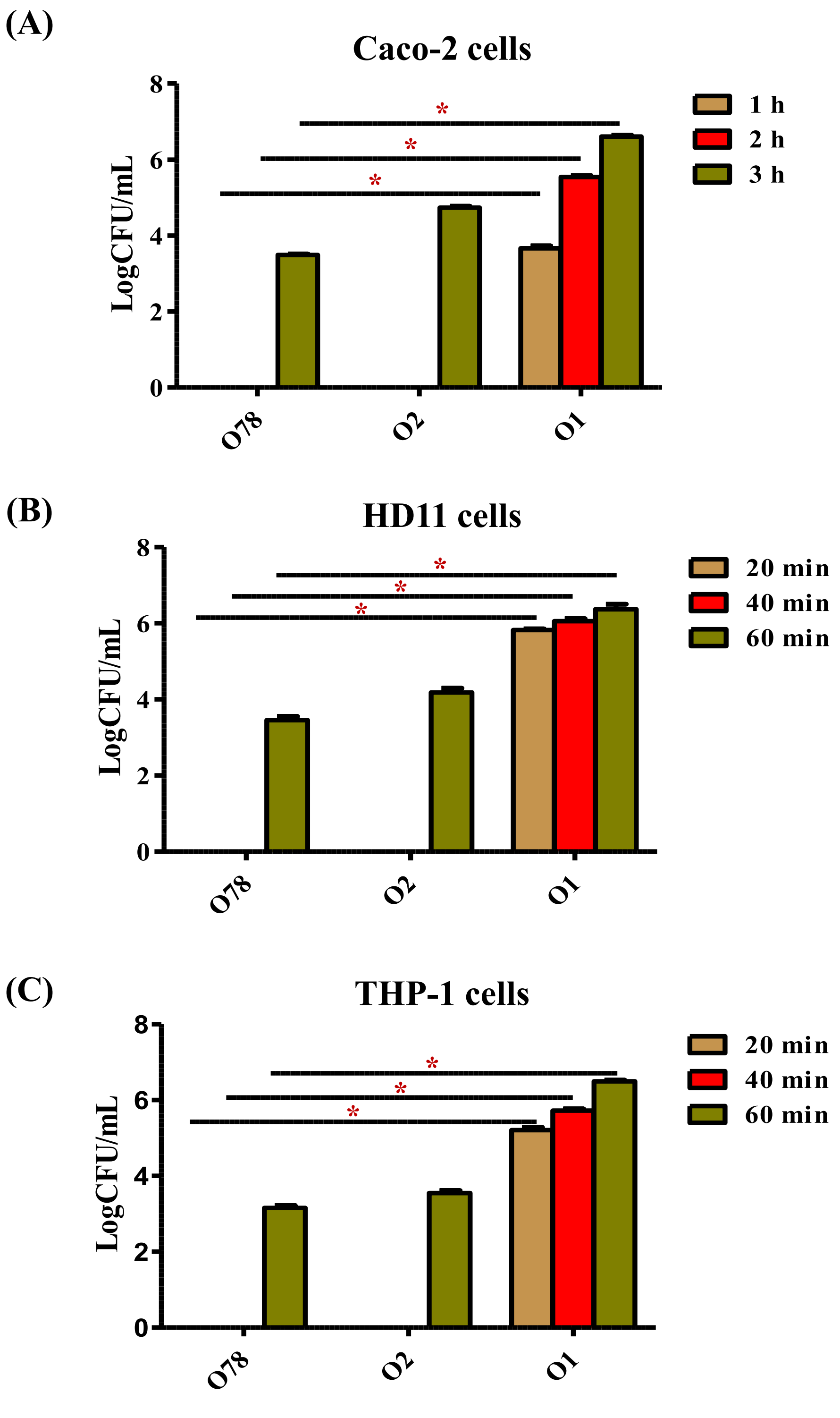
(A)
Caco-2 cells
LogCFU/mL
0
2
4
6
8
*
*
*
O78
O2
O1
1 h
2 h
3 h
(B)
HD11 cells
LogCFU/mL
0
2
4
6
8
*
*
*
O78
O2
O1
20 min
40 min
60 min
(C)
THP-1 cells
LogCFU/mL
0
2
4
6
8
*
*
*
O78
O2
O1
20 min
40 min
60 min

Figure S1. Effect of invasion time on the intracellular survival of APEC O78, O1, and O2 in **(A)** Caco-2, **(B)** HD11 and **(C)** THP-1 cells. Cells were infected at MOI:10 with APEC serotypes and were incubated for different time periods (Caco-2: 1, 2, and 3h; HD11/THP1: 20, 40, and 60 min) to allow bacterial invasion. Six hours following invasion, cells were lysed with 0.1% Triton X-100 and intracellular bacteria were quantified on LB agar plates. APEC O1 invades and survives intracellularly at significantly greater rate (comparable intracellular bacteria with 3 times less invasion time) than O2 and O78.*$P<0.01$.

Fig. S2

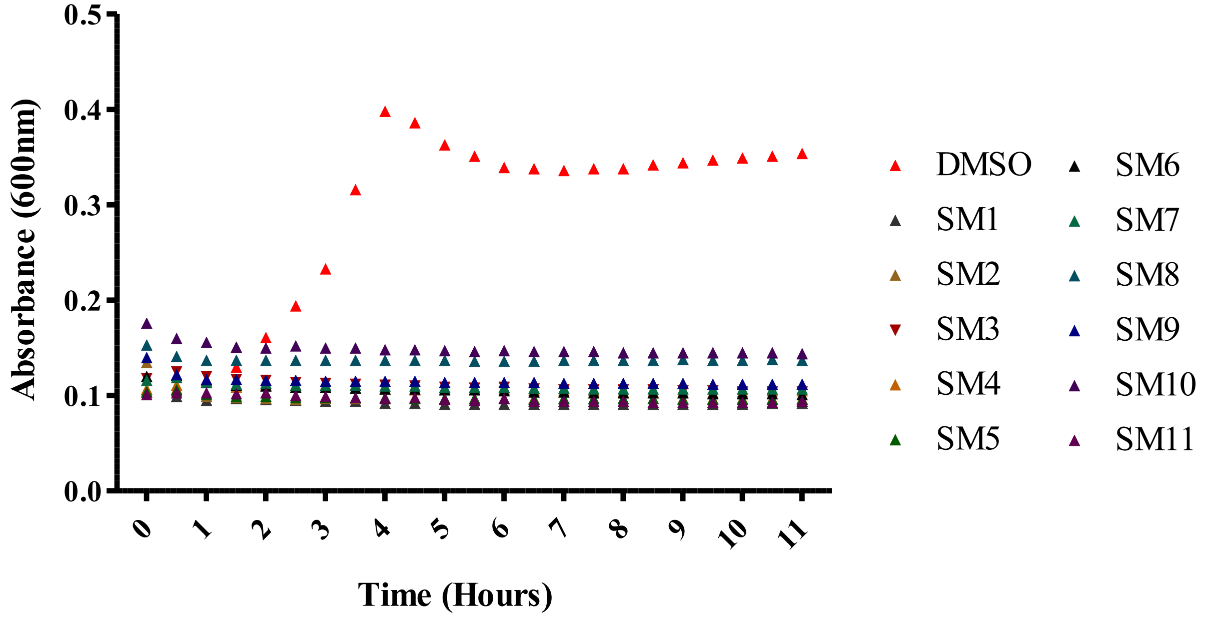

Figure S2. Effect of 11 cidal SMs to Rif^r APEC O78. All SMs inhibited the growth of Rif^r APEC O78 at respective MIC of wild type APEC O78 (**Table. S4**) as indicated by non-elevated OD.

Fig. S3

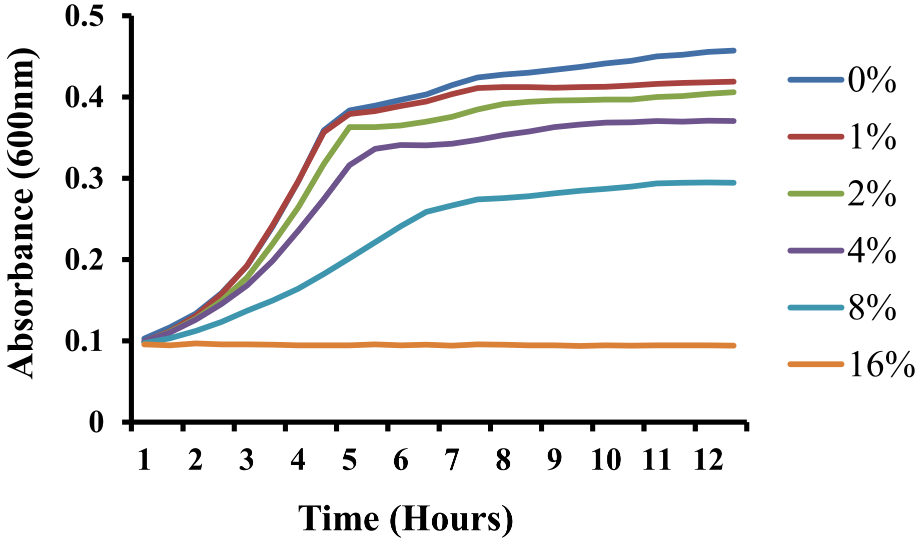

Figure S3. Antimicrobial activity of DMSO against APEC O78 in M63 media. APEC culture was incubated with different concentrations of DMSO followed by the monitoring of APEC growth for 12 h.

Fig. S4

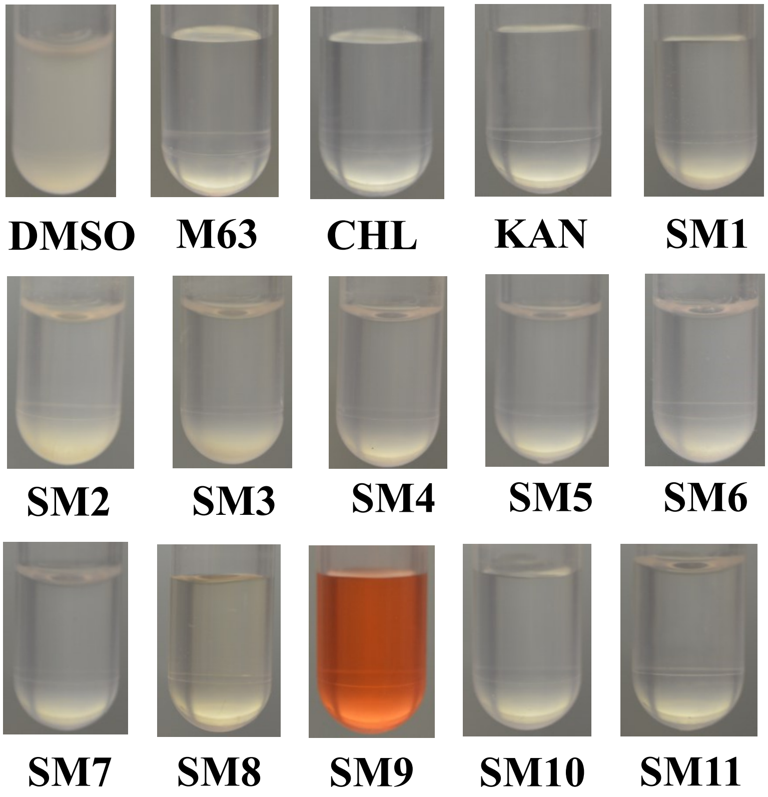

Figure S4. Resistance study in liquid media using sub-lethal concentration of SMs. APEC O78 was grown in the presence of 0.75X MIC of each SM in M63 media for 15 serial overnight passages and the bacteria were subsequently tested at the respective MIC. No turbidity was observed in the SMs treated cultures, while cultures treated with DMSO showed turbidity.

Fig. S5

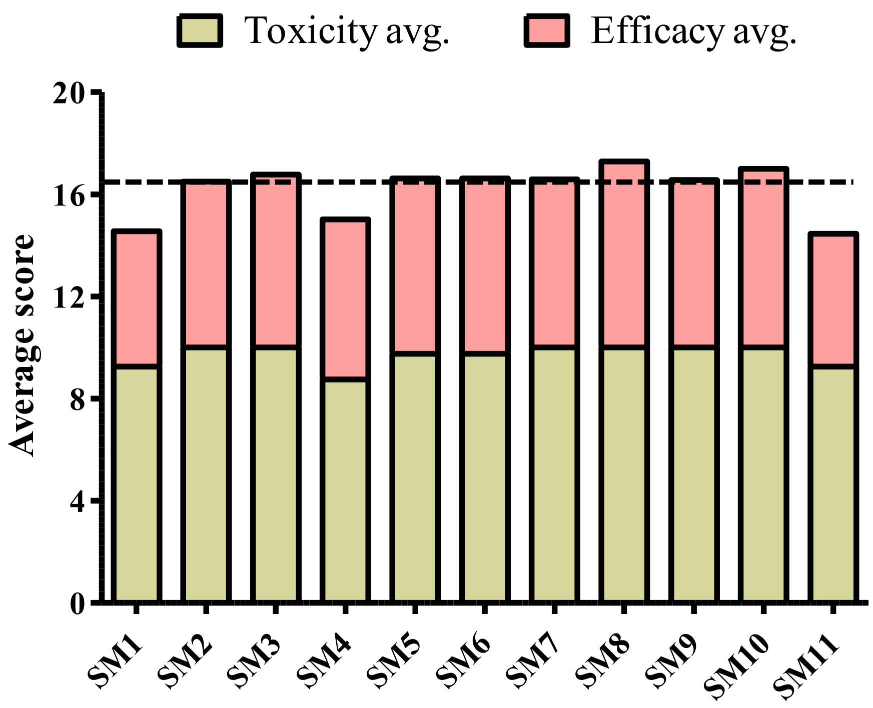

Figure S5. Prioritization of SMs based on their efficacy and toxicity. SMs toxicity (chicken & human cells, wax moth larvae) and efficacy parameters (MIC/MBC, spectrum, specificity, intracellular APEC clearance, wax moth survival, and reduction of biofilm embedded APEC) measured in this study were scored (1 to 10) and the scores were averaged as toxicity and efficacy average. SMs with average score above the demarcated line were regarded as potential anti-APEC therapeutics.
